# Data Visualizations to Support Health Practitioners’ Provision of Personalized Care for Patients With Cancer and Multiple Chronic Conditions: User-Centered Design Study

**DOI:** 10.2196/11826

**Published:** 2018-10-16

**Authors:** Uba Backonja, Sarah C Haynes, Katherine K Kim

**Affiliations:** 1 Nursing and Healthcare Leadership University of Washington Tacoma Tacoma, WA United States; 2 Biomedical Informatics & Medical Education University of Washington School of Medicine Seattle, WA United States; 3 Betty Irene Moore School of Nursing University of California Davis Sacramento, CA United States

**Keywords:** cancer care facilities, informatics, patient-centered care, patient-generated health data, precision medicine, visualization

## Abstract

**Background:**

There exists a challenge of understanding and integrating various types of data collected to support the health of individuals with multiple chronic conditions engaging in cancer care. Data visualization has the potential to address this challenge and support personalized cancer care.

**Objective:**

The aim of the study was to assess the health care practitioners’ perceptions of and feedback regarding visualizations developed to support the care of individuals with multiple chronic conditions engaging in cancer care.

**Methods:**

Medical doctors (n=4) and registered nurses (n=4) providing cancer care at an academic medical center in the western United States provided feedback on visualization mock-ups. Mock-up designs were guided by current health informatics and visualization literature and the Munzner Nested Model for Visualization Design. User-centered design methods, a mock patient persona, and a scenario were used to elicit insights from participants. Directed content analysis was used to identify themes from session transcripts. Means and SDs were calculated for health care practitioners’ rankings of overview visualizations.

**Results:**

Themes identified were data elements, supportive elements, confusing elements, interpretation, and use of visualization. Overall, participants found the visualizations useful and with the potential to provide personalized care. Use of color, reference lines, and familiar visual presentations (calendars, line graphs) were noted as helpful in interpreting data.

**Conclusions:**

Visualizations guided by a framework and literature can support health care practitioners’ understanding of data for individuals with multiple chronic conditions engaged in cancer care. This understanding has the potential to support the provision of personalized care.

## Introduction

### Background

About 1 out of every 4 people in the United States lives with multiple chronic conditions (MCC), which include cancer and other conditions such as hypertension, diabetes, and heart disease [1]. About 40% of individuals with cancer also live with 1 or more chronic conditions [2]. The attendant complexity and potential confounding factors in managing treatment-intensive illnesses such as cancer among individuals with MCC beg for personalized care approaches. Personalized care, as described in precision medicine and personalized medicine, involves the collaboration between health care practitioners (HCPs) and patients and considers the perspectives, experiences, and health-related data of the person receiving care [3]. A recent Cochrane review suggests that personalized care can support the physical and psychosocial health of individuals with MCC [3], which can include those engaged in cancer care. Person-generated health data (PGHD) such as symptoms, medication use, physical activity, and health goals are important information for personalizing care of MCC. Organizations, including the United States Department of Health and Human Services [4,5] and the Healthcare Information and Management Systems Society [6], recommend that PGHD be captured and used in decision making, care planning, and coordination. Furthermore, mobile technologies can support collection and access to PGHD to support the management of chronic conditions and personalized care [7-12].

Although PGHD is quite varied, it may be collected at a different velocity and magnitude and in different and nonstandard formats [11]. To be useful to HCPs, individuals with MCC (including those engaged in cancer care), and caregivers, the cognitive burden of understanding and synthesizing this information must be minimized, and the opportunity to make good decisions must be maximized. This is particularly pertinent in the care of individuals with MCC engaging in cancer care, which is the scope of the research described in this manuscript. Coordination of cancer care involves many people—the individual engaging in cancer care, their HCPs, caregivers, family, and health care staff—who need to integrate the large amounts of data; these data are used to support understanding of an individual’s health status, completion of health-related tasks, and care-related decision making [13].

Data visualization offers an approach to address this challenge of integrating and using large amounts of data collected to support the personalized care of individuals with MCC engaging in cancer care. Data visualizations are representations of data through the application of visual encodings (eg, position and color) [14-17]. Visualization can leverage a user’s cognitive strengths such as pattern recognition, and it helps them overcome their cognitive limitations including calculating and remembering strings of numbers. This can ultimately support understanding, task completion, and decision making. Visualizations that are designed with guidance from potential end users can be particularly valuable. User-centered design, within the field of human-centered design, is an approach to systems development that involves potential end users to understand their behaviors, tasks, and needs, among other factors [18,19]. User-centered design can help the designer fully render the users’ needs to improve the decisions that the visualizations are meant to support. User-centered design has been used previously in the development of visualizations [20,21] including visualizations of patient-reported outcomes [22,23].

The benefits of data visualizations—supporting understanding, task completion, and decision making—are especially critical in health-related settings such as cancer care facilities. In these settings, data are used to support important, critical, and time-restricted decisions that impact the health of individuals. Data visualizations are increasingly being incorporated into clinical care through integration of dashboards into health record systems. A recent review suggests that dashboards that integrate visualizations have the potential to support the cognitive work and decision making of intensive care unit clinicians [24]. However, there have been few examples of the effective use of person-generated data in personalized cancer care, particularly to enable shared decision making or care coordination [25]. A recent study found that patients with solid tumors who used a Web-based system to report symptoms experienced longer survival compared with usual care [26]. There is also little research on the value of visualizations within systems that integrate patient-generated data in cancer care. A pilot study conducted in Italy suggested that a dashboard that integrated remote monitoring and symptom-tracking data could be useful to HCPs and patients [27]. However, this work did not specifically evaluate the visualizations, and rationales for the visualization designs were not described. Therefore, a gap exists in the literature and practice regarding the development of informatics solutions that integrate and visualize person-generated data to support understanding and decision making regarding personalized cancer care among individuals with MCC.

### Prior Work

OnPoint is a mobile app developed by the authors to support care coordination for individuals with MCC [25,28,29]. Previous studies by the authors’ research group that were conducted to support heart failure and oncology patients resulted in the development of a mobile app that featured patient health goals, proactive symptom assessment, comprehensive medication list and medication reconciliation, and tracking for patient and caregiver use. On the basis of this prior work, researchers identified the need for visualization of data collected from the app and integrated into the electronic health record for communication to HCPs. This inspired the study described below.

### Study Purpose

The purpose of this study was to assess HCPs’ perceptions of and feedback regarding visualizations developed to support the personalized care of individuals with MCC engaging in cancer care.

## Methods

### Design, Time Frame, and Setting

This user-centered study took place from May to June 2017 at a large, urban academic medical center in the western United States.

### Recruitment of Participants

We sought 8 medical doctors (MDs) and registered nurses (RNs); a sample size considered adequate for this type of qualitative user-centered design study [30-32]. HCPs were either known to the researchers or identified by referral of HCPs who had participated in previous studies in the development of the OnPoint mobile app [25,28,29]. HCPs were eligible to participate if they were potential end users of a health information system to support cancer patients with MCC and HCPs currently providing care to cancer patients.

### Visualization Development

Paper mock-ups of data visualizations were developed by UB (author) guided by Munzner Nested Model for Visualization Design [17]. We applied all model constructs (italicized in the following paragraphs) except for the algorithm design construct, which is suited for software development rather than for our focus on presoftware development.

The *domain problem* addressed was the need to support the care of individuals with MCC engaging in cancer care—the problem addressed by the OnPoint mobile app [25,28,29].

For *operation and data type abstraction*, we identified operations (tasks) and data types from the previous studies [25,28,29]. These data types were blood pressure (mm Hg), weight (kg), blood glucose levels (mg/dL), medication adherence (medications not taken at the time or frequency as prescribed), and symptoms from the Canadian Oncology Symptom Triage and Remote Support (COSTaRS) practice guides [33,34].

For *visual encoding and interaction design*, mock-up encodings and designs were guided by literature on (1) visualizing data [14-17,35-44]; (2) health data visualization [23,45-47]; and (3) a mock patient persona and scenario [48,49]. The mock patient was a 56-year-old woman with uterine cancer and type 1 diabetes. She had recurrent work and personal constraints on Thursdays that interfered with taking medications as prescribed and managing her blood glucose levels. This patient also recently experienced weight gain due to fluid retention.

**Figure 1:**
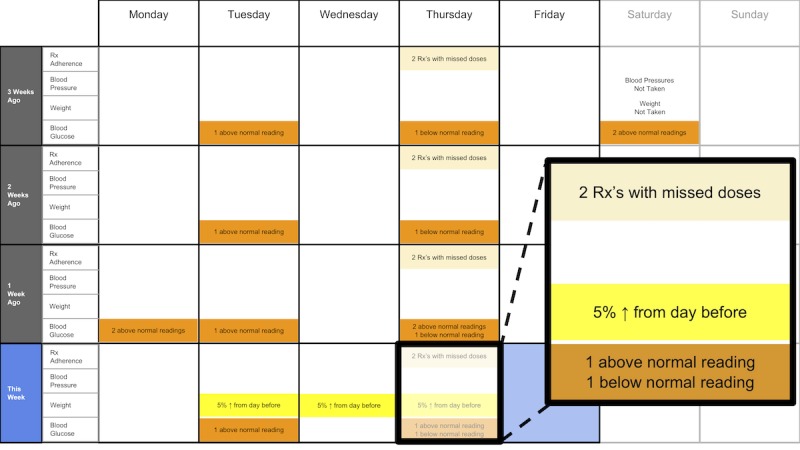
A 4-week overview of medication adherence, blood pressure, weight, and blood glucose.

**Figure 2:**
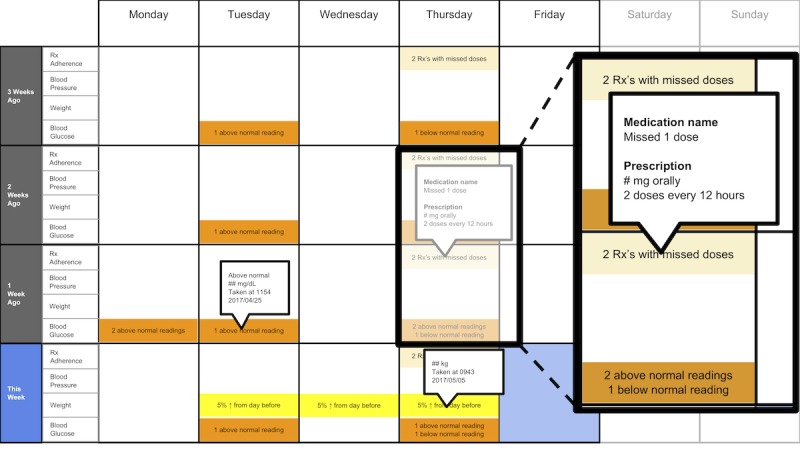
A 4-week overview of medication adherence, blood pressure, weight, and blood glucose with pop-ups providing details on demand.

**Figure 3:**
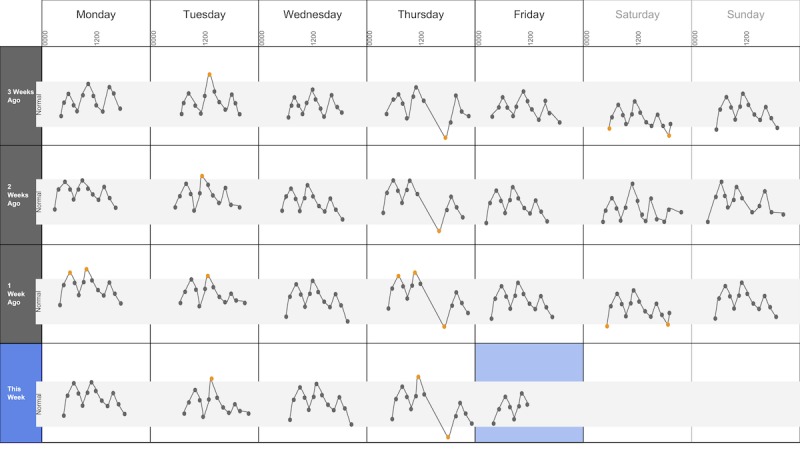
A 4-week view of blood glucose readings alone.

**Figure 4:**
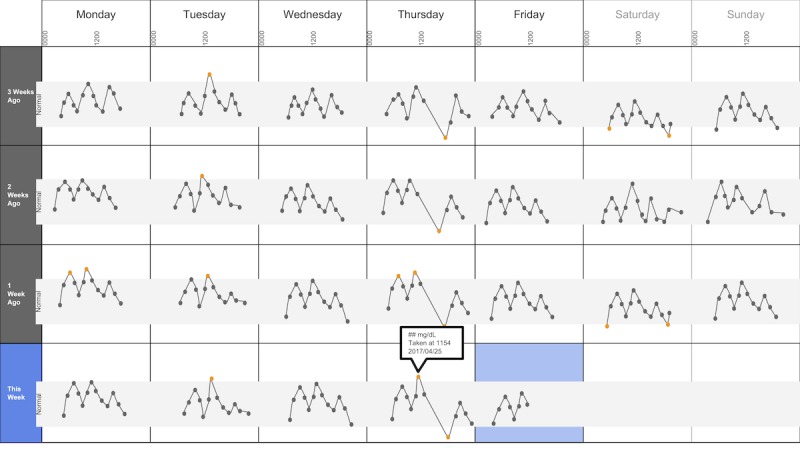
A 4-week view of blood glucose readings alone and with a pop-up providing details about a specific data point.

**Figure 5:**
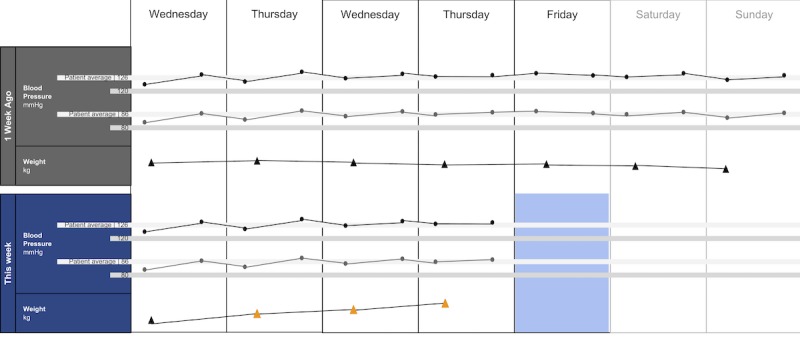
A 2-week view for blood pressure and weight.

**Figure 6:**
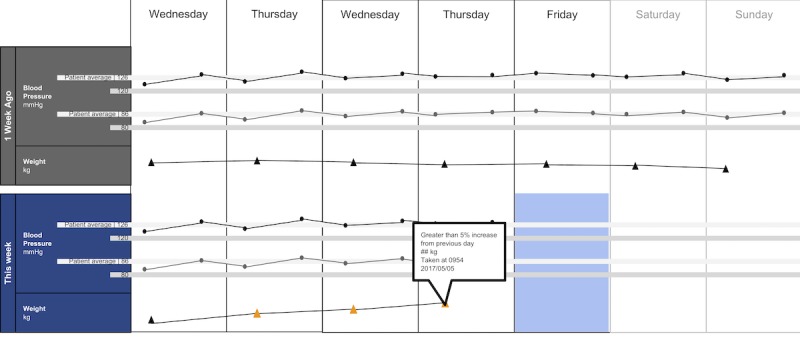
A 2-week view for blood pressure and weight with a pop-up providing details about a specific data point.

**Figure 7:**
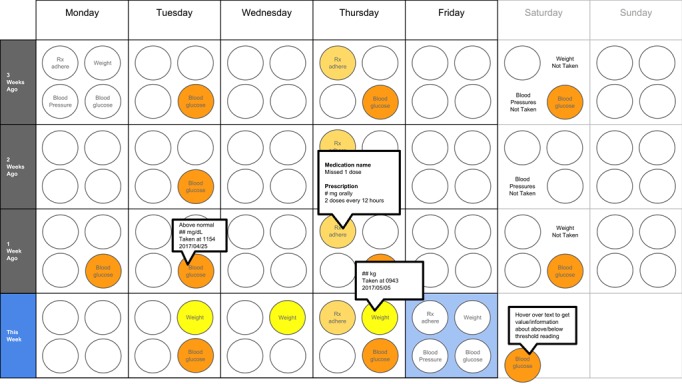
A 4-week overview circle view.

**Figure 8:**
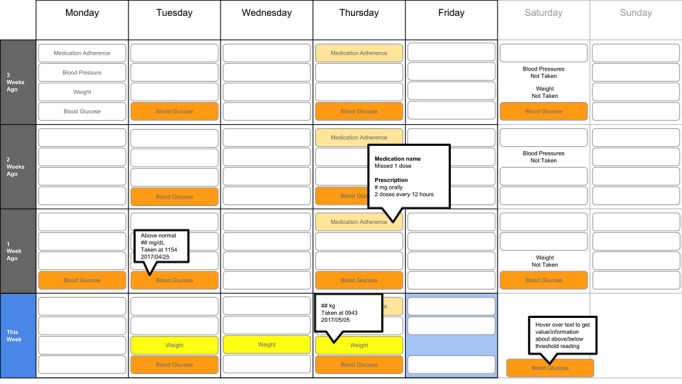
A 4-week overview all tab view.

**Figure 9:**
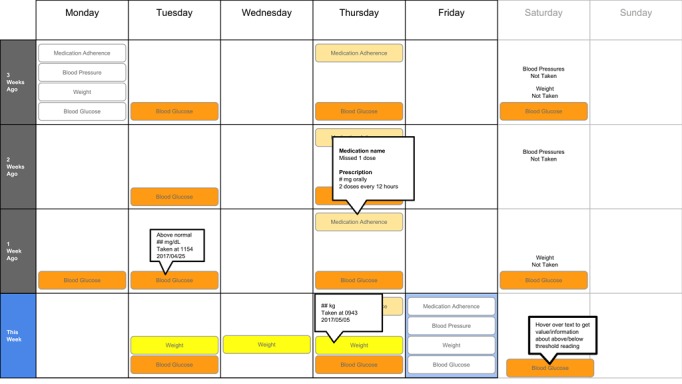
A 4-week overview filled tab view.

**Figure 10:**
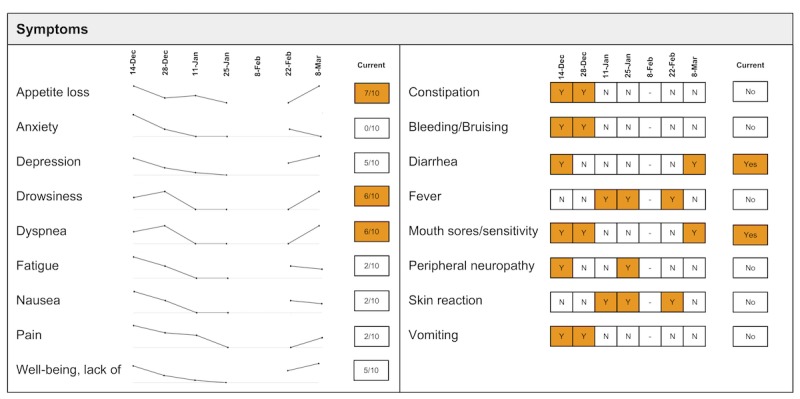
Visualization of patient-generated symptoms that are self-reported using the Canadian Oncology Symptom Triage and Remote Support (COSTaRS) protocols.

The following visualization mock-ups were created based on the nested model constructs: (1) a 4-week overview of medication adherence, blood pressure, weight, and blood glucose alone (Figure 1) and with pop-ups providing details on demand (Figure 2), (2) a 4-week view of line graphs indicating blood glucose readings alone (Figure 3) and with a pop-up providing details about a specific blood glucose reading (Figure 4), and (3) a 2-week view for blood pressure (Figure 5) and with a pop-up providing details about a specific blood pressure reading (Figure 6). In addition, 3 additional versions of the 4-week overviews of medication adherence, blood pressure, weight, and blood glucose were created (Figures 7-9). Finally, a visualization of self-reported symptoms generated from the COSTaRS protocols was developed (Figure 10).

Indication of target levels and ranges were shown with lines and colors. For example, in Figures 3 and 4, the gray bands indicate the target blood glucose range (80-130 mg/dL before a meal and <180 mg/dL 2 hours after the start of the meal [50]). In Figures 5 and 6, lines indicate (1) mock patient average systolic and diastolic blood pressure and target blood pressure (120/80 mm Hg [51]). Line graphs were purposefully chosen based on previous research indicating that position and color of dots on a chart (eg, individual glucose readings) can support quantitative interpretation [37-39,41]. Blue, orange, and yellow colors were used because they can be distinguished by individuals with color blindness [52-54].

### Measures and Procedures

This study applied user-centered design methods to engage potential end users early in the design process to understand their needs, priorities, and values. UB and SH conducted one-on-one interviews; participants were provided with pens, colored pencils, and markers and encouraged to draw and take notes on the paper visualizations as they reviewed them. They were also prompted throughout the interview to think aloud about what they saw and thought while reviewing the visualizations [55,56].

Interviews were conducted in 3 steps: (1) going through the scenario during which they thought aloud while viewing paper mock-ups and responding to prompts (see Textbox 1 for the scenario), (2) ranking of alternative visualizations of overview mock-ups, and (3) providing overall impressions and usefulness for care coordination. This step sequence was used to guide the participants in using the visualization as they might in their clinical practice, which can help elicit reflection from participants [48].

In step 1, participants first viewed an overview of visualization (Figures 1 and 2), then specific measures (Figures 3 and 4), then sought details on demand for those measures (Figures 5 and 6). This approach aligns with Shneiderman Visual Information Seeking Mantra of “Overview first, zoom and filter, then details-on-demand” [42]. While viewing the visualizations, participants explained what they saw, the impression of the patient, what additional information they would want included, what aspects supported their understanding of the patient, and what aspects were confusing.

In step 2, the researcher presented alternate versions of the 4-week overview (Figures 7-9). Ordering of the versions was varied from participant to participant so that the order in which the versions were presented did not influence responses. While viewing the alternate versions, participants described their overall impressions, aspects they perceived as helpful, and aspects they perceived as confusing. Then, the then researcher gave the clinicians the original 4-week overview (Figure 1) and asked participants to order this overview and the alternate versions from most helpful (ranked first) to least helpful (ranked fourth). Participants explained aloud their rationale for the ordering while sorting the versions.

In step 3, participants viewed the visualization that provided summaries of longitudinal patient-reported symptoms (Figure 10). Again, the participants were asked to describe their overall impression of the visualization, aspects that they perceived as helpful in understanding the patient, and aspects they perceived as confusing in understanding the patient. At the end of the interview, the researchers asked whether and how the visualizations could help personalize the care and asked for suggestions.

Interviews were recorded, transcribed, and supplemented by field notes taken by researchers during the interviews. This study was approved by the affiliated institutional review board. All participants provided verbal consent after receiving and reading the study consent form. Participants were provided with a US $50 Amazon gift card for engaging in the interview.

**Scenario used during one-on-one interviews with clinicians to elicit feedback about data visualizations. Information in brackets indicates actions by the researcher conducting the interview.**

1. You have arrived at the clinic before you start seeing patients. You want to see how your first patient of the day is doing. Her name is Deb Lee (age 56 years). Three weeks ago she completed chemotherapy for uterine cancer. She also has type 1 diabetes that was diagnosed in childhood.
2. You have done your typical chart review of Deb’s clinical data using the clinic’s electronic health record. After that chart review of electronic health record data, you want to see how Deb is doing at home. Recently the clinic started supporting patients in collecting data at home. Data include:
  - if the patient took her medications as prescribed
  - blood pressure (measured twice a day)
  - weight (taken once a day)
  - blood glucose (taken periodically throughout the day using a traditional finger-prick monitor)
3. These data collected by patients are provided to you first as 4-week summary. [Participant given Figure 1]
4. You want to know what’s going on with some of the data. You hover over several readings reading to get more information. After you hover, you get this visual. [Participant given Figure 2]
5. You are concerned about Deb’s blood glucose readings and want to see more details about her readings over the 4 weeks. You click on the most recent reading to get more information. After you click, you get this visual. [Participant given Figure 3]
6. You’d like to see some specific information about a specific data point. You hover over this orange dot. [Researcher points to dot on “This week,” Thursday at 12:00 pm]
7. After you hover over it, you get this visual. [Participant given Figure 4]
8. You are concerned about Deb’s weight readings and want to see details about her readings. You click on the most recent reading to get more information. After you click, you get this visual. [Participant given Figure 5]
9. You’d like to see some specific information about a specific data point. You hover over this orange dot. [Researcher points to dot for weight on “This week,” Thursday]
10. After you hover over it, you get this visual. [Participant given Figure 6]

### Analyses

Transcripts of participant interviews were analyzed independently by 2 researchers using directed content analysis [57]. Categories used to guide the development of codes and the content analysis were developed from the data and refined as described below. At first, 2 researchers independently coded 3 randomly selected sections of transcripts from different participants to identify themes. The unit of analysis was a distinct idea within a participant’s statement. After each of 3 rounds of independent coding, the researchers discussed how content was coded and any new themes that emerged for which codes needed to be added. After the third round, the researchers concurred that the codes adequately covered all themes, thus yielding the final codebook used for the remainder of transcript coding. Inter-rater reliability was calculated using an estimate of inter-rater reliability as described by Topf [58]. The agreement was 84.5%. The researchers discussed the discrepancies in coding and ultimately came to a consensus for a final inter-rater agreement of 100%. Researchers then independently coded the transcripts using NVivo (v11.4.1, QSR International, Melbourne, Australia). Rankings for preferences of the 4 versions of the overview visualization (Figure 2; Figures 7-9) were tabulated, and mean ranks and SD were calculated for each overview version.

## Results

### Participants

A total of 8 HCPs participated in the interviews. Out of these, 4 were MDs; 1 was a pain management specialist (participant MD1), and 3 were oncologists (participants MD2, MD3, and MD4), and 4 were RNs (participants RN1, RN2, RN3, and RN4). Each participant provided care to cancer patients in the cancer center. Interviews lasted for approximately 25 to 42 min.

### Themes

We identified 7 themes. Of these, 2 themes were not directly relevant to the visualizations; therefore, for the purpose of this paper, we report the following 5 themes: data elements, supportive elements, confusing elements, interpretation, and use of visualization. See Table 1 for descriptions and specific content regarding the themes.

**Table 1:** Themes identified from interviews with health care practitioners while evaluating visualizations to support cancer care of an individual with multiple chronic conditions.

Theme	Description	Specific content regarding the theme
Data elements	Existing or future or potential data elements (eg, weight, blood pressure, medication adherence, and symptoms)	data useful for specific role in cancer care included weight and medications (Figures 1,5, and 6) and the list of symptoms (Figure 10) [MD^a^1 and RN^b^2] data less critical for some given job roles included blood glucose measures (Figures 3 and 4) [RN4 and MD4] suggestions for additional data elements or information included additional measures such as heart rate [MD1 and RN4], temperature [RN1 and RN2], body mass index [MD3], lab values [RN1], meal times or what eaten [RN1, RN2, MD3, and MD4], physical activity engagement [RN1], sleep [MD1], swelling [MD2], symptoms that may be particular or specific to certain cancer therapies [MD2, RN2, and MD3] patient-identified symptoms [RN2 and MD3] a legend defining the symptoms [MD1] meaning of the symptom scale ratings [MD1] reasons for missed medications [MD2, MD3, and RN1] values and description of the normal values and ranges for blood glucose and blood pressure [MD2] goals of care [RN1] treatments [RN1] patient-reported reasons for abnormal values [RN2]
Supportive elements	Aspects of the visualization that supported the participant’s understanding of the patient or that they thought were helpful	the color orange drew attention and helped participants find data points or patterns in the data that might require attention or indicate something abnormal more easily [MD1, MD3, MD4, RN1, RN3, and RN4] icons of different shapes in Figures 5 and 6 helped participants follow the line graph progression [MD1, MD2, MD4, and RN2] gray bands indicating normal ranges (Figures 3-6) helped identify abnormal data points [MD2, MD3, RN1, and RN4] calendar format and line graphs were helpful because clinicians are accustomed to them [MD1, RN1, RN2, and RN4], are used in practice [MD1 and RN1], and help see trends [MD1-MD4 and RN1-RN4] having details on demand was helpful [MD1-MD4 and RN1-RN4] and does not to lead to overpopulation of data within the visualization [MD3] suggestions for additional supportive elements included a pop-up with a numeric scale for normal ranges [MD4] or an indication of how the normal range was derived [MD3]
Confusing elements	Aspects of the visualization that the participant does not understand or finds confusing or unhelpful	Figure 1: Unsure if blank spaces indicated that measurements were normal or not taken [MD2, MD3, and MD4] Figures 1 and 5: Unclear about how the weight increase was calculated [MD2, MD3, and RN4] Figure 7: Circles confusing or overwhelming [MD2, MD4, RN2, RN3, and RN4] Figure 8: Unsure if the empty rectangles indicated normal readings or no measures taken that day [RN2, RN3, RN4, and MD4] Figure 10: Lack of clarity about the meaning of the numeric scale [RN3 and RN4]; unsure about threshold values for the numeric scores that led to values being highlighted in orange or whether the thresholds were the same across all symptoms [RN1]; miniature line graphs hard to interpret [MD1, MD2, MD4, and RN3]; and hard to understand, compare, and interpret the 2 different ways of quantifying symptoms [RN1, RN2, and RN4]
Interpretation	Information obtained or conclusions drawn about the patient from the visualization	Figures 1 and 2: Used calendar view to identify issue of missed medications of Thursdays [MD1-MD4, RN2, and RN4] Figures 3 and 4: Dips and peaks in the blood glucose line graph helped identify instances of hypo- or hyperglycemia [MD1 and MD2] or hypothesize if patient had well-controlled blood glucose [MD7 and MD8]; time indications at the top of the graph or pop-up helped hypothesize how meals may relate to dips and peaks [MD2, MD4, and RN1]
Use of visualization	Ways the participant would or could use the visualization	visualizations could help clinicians gain an understanding of patient outside the clinic [MD1 and MD2], help them prepare specific questions regarding Thursdays [MD1-MD4, and RN4] and the cause of rapid weight gain [MD2], discuss the patient’s symptom experiences or management [RN4 and MD4], make clinic time more efficient [MD1 and MD2] visualizations could help patients remember health experiences [MD2] and empower patients to engage in health management [MD4]. Together clinicians and patients could use visualizations to support personalized cancer care [MD1-MD4, and RN1-RN4], facilitate interactions that focused more on the patient and their specific needs [MD1, MD2, RN2], and better guide conversations between clinicians and patients [RN3, RN4, MD3, and MD4]

^a^MD: medical doctor.

^b^RN: registered nurse.

### Data Elements

There were several data elements within the visualizations that participants indicated as useful and supportive for their collaborations with patients. Participants noted the usefulness of measures and behaviors portrayed in Figures 1-6 (eg, weight) and symptoms portrayed in Figure 10 (eg, pain levels). Certain data were noted as being less relevant given their roles (see Table 1). Participants indicated several additional data elements that could be helpful (see Table 1). This included symptoms identified by patients as relevant or important (n=2). For example, MD3 suggested patient-driven modifications of the symptoms list:

Can we plan another category that I want the patient to monitor? For example, if they’re having bleeding-vaginal bleeding-can they use the category of vaginal bleeding to show me...?

MD3

### Supportive Elements

Participants described several visual elements that supported their understanding of the patient. The elements included color, the calendar format, use of line graphs, and the ability to get “details on demand.” All participants (n=8) stated that color supported their understanding of the data. Several indicated that icons helped differentiate graphs (n=4) and that the gray bands indicating normal ranges helped identify abnormal data points (n=4). Participants stated that the calendar format and line graphs were helpful because they are accustomed to them (n=4), are used in practice (n=2), and help them see trends (n=8):

[The line graph] gels with what practitioners could be used to...You don’t want to have something too novel where you have some bizarre bar graph or some kind of odd, interesting pattern that’s in 3D...that people haven’t seen.

MD1

Three participants stated that the calendar format allowed them to see trends such as missed medications on Thursdays (RN1, RN2, and RN4):

...you see a pattern...that helps you identify that there is a regimen and that there’s a schedule...it enables you to see something missed in the pattern by seeing the...[entire] month.

RN4

All participants (n=8) reacted positively to “details on demand” features such as hovering over a data point to get a pop-up with detailed information:

...it’s good that it [the visualization] doesn’t overpopulate the numbers right there and then because I mean I would just be overwhelmed with actual numbers, so this hovering thing is really good.

MD3

### Confusing Elements

There were several visual elements in the 4-week overviews that participants found confusing. These included not understanding the meaning of blank spaces in Figure 1 (n=3) or Figure 8 (n=4), being confused or overwhelmed by the circles in Figure 7 (n=5), and lacking clarity about how the weight increase was calculated for Figures 1 and 5 (n=3).

Several participants noted issues with interpreting visualizations for patient-reported symptoms (Figure 10). This included lack of clarity about the meaning of the numeric scale, threshold values for the numeric scores that led to values being highlighted in orange, and whether the thresholds were the same across all symptoms (n=3). Participants also found it difficult to interpret the miniature line graphs (n=4) and to understand, compare, and interpret the 2 different ways of quantifying symptoms (n=3).

### Interpretation

All participants (n=8) used visual elements to interpret data—finding patterns and viewing trends—to support understanding and decision making. They identified missed medications including the pattern of missed medications on Thursday using the calendar views (n=6). When seeing the pattern on Thursdays, participants were prompted to think about what could cause the patterns:

I wonder what’s going on Thursdays that she always forgets the medications.

MD3

Identifying this pattern supported RN4’s decision making to investigate the cause of the pattern:

I’m not sure why [she is missing her medications consecutively on Thursdays] so you would have to find out why is she missing her drugs on Thursday.

RN4

MD4 similarly described how using the visualizations supported understanding of the patient, reasoning about what might be causing abnormal readings, and ruling out potential causes:

...she just is not taking her medications for some reason...I can use the visualization and the colors to figure out some of her difficulties...[about] why she may not be adherent with her health [behaviors]and medications.

MD4

RN4 echoed how the visualizations could facilitate clinician reasoning, stating that a clinician could postulate about what might be causing the issues on Thursdays by bringing in symptoms and other data:

...you could really get I think a good picture.

RN4

Trends in line graphs helped participants interpret temporal glucose, blood pressure, and weight data (n=5). Participants reflected on several weeks’ worth of data, comparing normal and abnormal points over time as well as visual elements indicating missed medication, to formulate whether they believe the patient had well-controlled blood glucose (n=2). For example, MD4 stated:

[if the medication being missed is] related to her insulin...and her blood sugars aren’t controlled, [then] the general impression probably is that her blood sugars aren’t controlled and her diabetes isn’t controlled.

MD4

Participants viewed trends across different measures to infer relationships between measures. When viewing Figures 5 and 6, MD1, MD2, RN1, and RN4 viewed the line graph trend for blood pressure, guided by the gray bands indicating normal readings and color coding of the data points, to inform their reasoning about what might have caused blood pressure to stay within normal range but weight to increase (as indicated by the line graph trend and color-coded data points). Participants used the calendar structure to see if weekly patterns were consistent:

I also see that the same kind of pattern I’ve seen on this very day and the week before.

MD3

### Use of Visualizations

Participants stated that visualizations could help gain an understanding of the patient outside the clinic and prepare specific questions to facilitate discussions with the patient about their self-management outside the clinic. This included discussing circumstances on Thursdays that made health management challenging (n=5), asking questions to help investigate the cause of rapid weight gain (n=1); and understanding symptom experiences or management (n=2). MD2 stated that the visualizations provide insights that “might open up a door to other questions that you normally wouldn’t ask if you didn’t [see trends].”

In addition, participants noted that visualizations could be helpful for patients. Having the visualizations during clinic visits could help patients remember symptoms; MD2 stated that the visualizations provide “another way to understand if the patient had any symptoms but forget to mention [them] to us or we forgot to ask [about them] during the clinic visit.” Visualizations could also support patients feeling empowered; MD4 stated that a visualization tool could empower patients to engage in health behaviors such as taking medications and “be more aware of their symptoms” regularly.

All participants (n=8) mentioned the use of visualization to personalize visits with patients. Visualizations helped them identify issues specific to the patient that needed to be addressed, making interactions more focused on the patient and their specific needs (n=3). Visualizations could also give a clearer and focused picture of the patient, their health status, and needs that can better guide conversations and interactions with patients (n=4):

I think it would cause us to get a good picture, get a fast picture, evaluate that with the patient so we don’t walk into an assumption, but dive a little bit quicker if we needed to.

RN4

MD3 stated that the visualization could personalize visits by bringing “attention to the important things” and focus “conversations with the patient directly to what’s the issues or the problems that now I see [using the visualizations]...” RN4 stated that a benefit of the visualizations through personalizing visits with patients could be earlier identification of issues “rather than waiting until things [snowball].”

### Ranking of Overview Visualizations

Participants varied in their preferences for the 4-week overviews. On average, participants ranked Figure 2 as most helpful (mean 1.8 [SD 1.2]) and Figure 7 least helpful (mean 3.9 [SD 0.4]; see Table 2).

**Table 2:** Participant rankings of the four 4-week overview versions.

Figure version	MD^a^1	MD2	MD3	MD4	RN^b^1	RN2	RN3	RN4	Ranking^c^, mean (SD)
Figure 2	1	2	1	1	4	1	3	1	1.8 (1.2)
Figure 9	2	1	2	3	2	2	2	2	2.0 (0.5)
Figure 8	3	3	3	2	1	3	1	3	2.4 (0.9)
Figure 7	4	4	4	4	3	4	4	4	3.9 (0.4)

^a^MD: medical doctor.

^b^RN: registered nurse.

^c^Mean rankings (and SD) across all participants for each version and ordered from most helpful (closest to 1) to least helpful (closest to 4).

## Discussion

### Principal Findings and Comparison With Prior Work

In our user-centered design study, we found that MD and RN participants’ understanding of physiological and symptom data for individuals with MCC engaged in cancer care was supported by visualizations we developed by applying a visualization framework and relevant literature. Both MD and RN participants found that various visual encodings such as color, and familiar presentation such as calendar formats and line graphs supported their interpretation of the presented data. This research used foundational knowledge in data visualization in a novel way to develop visualizations that both MD and RN participants found helpful and effective in integrating various health-related data. Our MD and RN participants also noted the potential usefulness of the visualization in supporting personalized care. This user-centered design study offers findings from potential clinician users of the output of patient-generated health data from the OnPoint app. These results will be used specifically to inform the integration of visualizations into OnPoint system in the next phase of the project.

We demonstrated that using paper prototypes early in the design process allowed us to engage potential end users, gather useful insights, and explore suggested changes efficiently before investing in technical resources to build the system. We found that there were similar reactions by MDs and RNs to the visualizations. For example, all MD and RN participants found bands representing normal ranges and details on demand to be helpful, and they perceived the visualizations as helpful in providing personalized care. Both MDs and RNs reported that color helped them pick out important data points and that the blood glucose graph dips and peaks helped them think about what might have caused abnormal readings. This suggests that careful design of visualizations that incorporate fundamental guidance of data visualization can support a wide range of users. Although personalization and customization of a visualization interface based on different users’ needs could increase usefulness and usability [59-61], it is possible to minimize the extent to which visualization versions differ when they are designed thoughtfully and purposefully.

Although we cannot assume that an interactive tool incorporating visualizations for use by individuals with MCC engaged in cancer care would necessitate the same design as a tool for clinicians, we do believe that this study offers a starting point for features to consider for users who are patients.

This study has the potential to inform the growing domain of research in integrating visualizations into informatics solutions that support personalized patient care [45,62-71]. This includes work on integrating home monitoring data for individuals engaging in cancer care [27] as well as health-related quality of life data for prostate cancer care [72].

Our study findings are congruent with guidance and best practices described in the visualization literature. Both MD and RN participants noted that color helped them see patterns in the data or pick out data that require attention, congruent with work described by Ware [44]. They also were able to use the line graphs to identify meaningful patterns in the data; this aligns with recommendations based on work by Cleveland and McGill [37-39] and Mackinlay [41]. In particular, position rather than other data encodings (eg, area) supports more accurate interpretation of the data being represented by the encoding. Although we did not compare our line graphs with other graph types in this study, participants responded positively to our design choice that was guided by the data visualization literature.

### Implications for Developing Health-Related Visualizations

On the basis of the findings from our study and the current literature of integrating visualizations into clinical care, we propose the following design recommendations: (1) applying knowledge from both health informatics and visualization domains to guide the creation of visualizations and (2) applying previous research can facilitate the development and testing of systems that integrate health data visualization. First, using Munzner Nested Model for Visualization Design [17] supported the design process by making it efficient, and it can facilitate integration of our findings with other research using the same model [73].

Second, providing users with options on how to visualize the same data may support use of the visualization. In our study, we found that among the 4-week calendar view options, there was not 1 that was consistently favored. Following 1 of Nielsen usability heuristics—flexibility and efficiency of use—visualization tool developers could allow users to customize how data and information are displayed [59-61].

Finally, engaging potential end users early in the design ideation process was feasible and insightful. To minimize time and burden on HCP participants, researchers can carefully develop study protocols so they can maximize opportunities for participants to provide insights such as using mock patient personas and scenarios to guide eliciting feedback about mock-ups. Using personas and scenarios is advocated within the human-computer interaction domain [48,49], and it has been used to support the development of health informatics tools [74,75].

### Limitations

There were limitations to our study. Our sample was limited to MDs and RNs; these visualizations could be useful to other HCPs supporting individuals with MCC engaging in cancer care, such as care coordinators, dieticians, pharmacists, and social workers. In addition, inputs from patients themselves and their family members must be collected to understand their informational needs. This study was conducted at a single cancer center; therefore, it has limited generalizability to other cancer centers. Mock-ups were on paper rather than on a device that a clinician would use to view visualizations in practice (eg, computer tablet and desktop computer). The data visualization literature used to guide the development of our mock-ups has not been tested extensively and empirically within health-related apps for cancer care; our work can support the building of evidence regarding the application of the visualization literature within this health domain. Although the visualizations are intended to be delivered via the electronic health record, we did not explicitly address how this might be accomplished. This work will be pursued in a future phase.

### Conclusions

This study suggests that visualizations guided by a framework and literature can support HCPs’ understanding of data to support personalized cancer care for individuals with MCC. By integrating health informatics and visualization literature and applying user-centered design methods, we were able to develop and elicit feedback on visualizations for health-related data including person-reported data. Future research could apply these methods toward the development of visualizations to support the care of other populations and the development of functional systems integrated into clinical and personal health care.

## Abbreviations

COSTaRS: Canadian Oncology Symptom Triage and Remote Support
HCPs: health care practitioners
MCC: multiple chronic conditions
MD: medical doctor
PGHD: person-generated health data
RN: registered nurse
